# The effect of milrinone on mortality in adult patients who underwent CABG surgery: a systematic review of randomized clinical trials with a meta-analysis and trial sequential analysis

**DOI:** 10.1186/s12872-020-01598-8

**Published:** 2020-07-08

**Authors:** Yu-shan Ren, Lan-fang Li, Tao Peng, Yu-jun Tan, Ying Sun, Guo-liang Cheng, Gui-min Zhang, Jie Li

**Affiliations:** 1 Shandong New Time Pharmaceutical Co, Ltd., Linyi, China; 2 National Engineering and Technology Research Center of Chirality Pharmaceutica, Lunan Pharmaceutical Group Co, Ltd., Linyi, China; 3 State Key Laboratory of Generic Manufacture Technology of Chinese Traditional Medicine, Lunan Pharmaceutical Group Co, Ltd., Linyi, China

**Keywords:** Milrinone, Meta-analysis, Mortality, Postoperative outcomes

## Abstract

**Background:**

As an inodilator, milrinone is commonly used for patients who undergo coronary artery bypass graft (CABG) surgery because of its effectiveness in decreasing the cardiac index and mitral regurgitation. The aim of this study was to perform a systematic review and meta-analysis of existing studies from the past 20 years to evaluate the impact of milrinone on mortality in patients who undergo CABG surgery.

**Methods:**

We performed a systematic literature search on the application of milrinone in patients who underwent CABG surgery in studies published between 1997 and 2017 in BioMed Central, PubMed, EMBASE, and the Cochrane Central Register. The included studies evaluated milrinone groups compared to groups receiving either placebo or standard treatment and further compared the systemic administration.

**Results:**

The network meta-analysis included 723 patients from 16 randomized clinical trials. Overall, there was no significant difference in mortality between the milrinone group and the placebo/standard care group when patients underwent CABG surgery. In addition, 9 trials (with 440 randomized patients), 4 trials (with 212 randomized patients), and 10 trials (with 470 randomized patients) reported that the occurrence of myocardial infarction (MI), myocardial ischemia, and arrhythmia was lower in the milrinone group than in the placebo/standard care group. Between the milrinone treatment and placebo/standard care groups, the occurrence of myocardial infarction, myocardial ischemia, and arrhythmia was significantly different. However, the occurrence of stroke and renal failure, the duration of inotropic support (h), the need for an intra-aortic balloon pump (IABP), and mechanical ventilation (h) between these two groups showed no differences.

**Conclusions:**

Based on the current results, compared with placebo, milrinone might be unable to decrease mortality in adult CABG surgical patients but can significantly ameliorate the occurrence of MI, myocardial ischemia, and arrhythmia. These results provide evidence for the further clinical application of milrinone and of therapeutic strategies for CABG surgery. However, along with milrinone application in clinical use, sufficient data from randomized clinical trials need to be collected, and the potential benefits and adverse effects should be analyzed and reevaluated.

## Background

In 2017, the World Health Organization (WHO) reported that nearly 17.7 million people die of cardiovascular diseases (CVDs) every year, accounting for 31% of all global deaths. Coronary artery disease (CAD) refers to the class of diseases of vascular stenosis or obstruction caused by coronary artery atherosclerotic lesions, resulting in myocardial ischemia, hypoxia or necrosis and including stable and unstable angina, myocardial infarction (MI), and sudden cardiac death [1]. Furthermore, CAD can cause serious complications due to multiple risk factors, such as a heart attack, damaged heart muscle, and an irregular heartbeat, and can result in sudden death [2–4] . At present, coronary artery bypass grafting (CABG) surgery is the primary strategy for CAD treatment [5–9]. CABG surgery is a surgical procedure in which vascular access between the root of the ascending aorta and the distal end of the lesion site is established to make blood bypass the coronary artery lesion site, flow to the distal end of the coronary artery stenosis or obstruction, and reach the ischemic myocardium, thus improving coronary perfusion and increasing myocardial oxygen supply [10–12]. Although CABG surgery has been reported to be associated with low costs, superior outcomes, and particularly short-term mortality [13–16], multiple complications, such as MI, myocardial ischemia, arrhythmia, stroke, and acute renal failure (ARF), are impossible to ignore and are still concerning to researchers and clinical doctors [7, 17–20], To minimize the occurrence of postoperative complications, pre- and/or postoperative medicinal applications, such as phosphodiesterase (PDE) III inhibitors, have been the primary strategies to date [21–23].

By reducing the inactivation of cyclic adenosine phosphate (cAMP) in cardiomyocytes, PDE III inhibitors enhance myocardial contractility and produce positive inotropic effects [24, 25]; a higher concentration of cAMP results in contractility, increasing myocardial tissue and the vasodilatory effect on vascular smooth muscle [26, 27]. Milrinone, a PDE III inhibitor, is primarily used after open-heart surgery because it can avoid cardiopulmonary bypass [28], enhance cardiac contractility [29], prevent vasospasm [30], and ameliorate low output syndrome (LOS) [31]. However, recent studies have demonstrated that the efficacy and safety profile of milrinone remains controversial, although it has been implemented in several guidelines [32, 33]. In some studies on cardiac surgeries, a tendency for an increased mortality rate and incidence of arrhythmia has been found in milrinone groups compared with control groups [34, 35]. However, another study evaluating milrinone for acute heart failure treatment revealed that milrinone might be safe and effective [36]. All contradictory outcomes resulted from the limited number of included patients [34] and the lack of key methodological criteria [37] not based on previously published protocols [35]. No studies have assessed the incidence of postoperative complications.

To avoid bias results from any unclear risk of bias that were included, our objective was to conduct a systematic review and meta-analysis of existing randomized controlled trials (RCTs) and to assess mortality between milrinone-treated patients and patients receiving placebo/standard care. The incidence of postoperative complications, such as MI, myocardial ischemia, arrhythmia, stroke, and acute kidney injury (AKI), was estimated simultaneously.

## Methods

### Search strategy

The search strategy aimed to include any RCTs conducted among adult patients who underwent CABG surgery and were treated with milrinone and in which these patients were compared to those treated only with placebo/standard care. A pertinent study search was independently conducted in BioMed Central, PubMed, Embase, and the Cochrane Central Register (all searches updated in November 2017) by 3 trained investigators [Lan-fang Li, Guo-liang Cheng, and Ying Sun]. We searched database for randomised trials through coronary artery bypass grafting (CABG) in the treatment of left main coronary artery disease with pre- and/or postoperative milrinone applications using the search terms “coronary artery bypass operation”, “coronary artery bypass grafting”, “randomised”, “randomized”, postoperative milrinone, or preoperative milrinone. No language restrictions were imposed, and non-English-language articles were translated before analysis.

### Study selection

References retrieved using the literature searches and databases were screened. When potentially pertinent studies were found, complete articles were retrieved. The inclusion criteria were as follows: patients randomly allocated according to treatment, groups receiving milrinone compared with groups receiving placebo/standard care with no restrictions in terms of dose or time of administration, CABG surgery performed in adult patients, and information provided on primary outcomes (endpoint). The exclusion criteria were as follows: lack of outcome (mortality) data, duplicate publications, animal experimental studies, articles published as abstracts only, and pediatric populations. Three investigators independently assessed compliance with the selection criteria and selected studies for the final analysis; divergences were resolved by consensus, and if issues persisted, the reference was evaluated by 4 investigators independently.

### Data extraction and study characteristics

The following details were independently extracted from the retrieved studies by 4 trained investigators: number of patients, surgical type, clinical setting, milrinone dosage, treatment duration, follow-up, mortality, and operative complications (such as MI, myocardial ischemia, arrhythmia, stroke, and AKI). The primary endpoint of the current analysis was mortality. Additionally, MI (per author definition), acute renal failure (per author definition), myocardial ischemia, arrhythmia, stroke, AKI, mechanical ventilation, lengths of intensive care unit and hospital stay were the secondary endpoints.

### Quality assessment

The included trials were assessed according to the Cochrane Collaboration methods to evaluate the risk of bias and the internal validity by 3 independent reviewers.

### Data analysis and synthesis

RevMan (Review Manager, version 5.2, Nordic Cochrane Center, Cochrane Collaboration, Copenhagen, 2012) and Stata (Stata Statistical Software: release 13, StataCorp LP, College Station, Texas) were utilized to analyze data extracted from the selected articles. A Q-test was applied to measure the statistical heterogeneity, and I^2^ was used as a quantitative measure of the degree of heterogeneity. The date of mortality was estimated to compute the individual and pooled relative risk (RR) with a 95% confidence interval (CI) by means of the Mantel-Haenszel method. The presence of heterogeneity across trials was also evaluated, with I^2^ < 25% indicating no significant heterogeneity when the fixed-effects model was used. In contrast, in the case of moderate or substantial heterogeneity (I^2^ > 25%), a random-effects model was used. Funnel plots were used to explore the small-study risk of bias by analytic appraisal based on Peters’ regression asymmetry test. Meta-regression analyses were performed to investigate sample size, mean number of grafts, mean number of arterial grafts, mean pump time, mean AoXclamp time, mean preop left ventricular ejection fraction (LVEF), pre-op drugs, post-op inotropes, pre-op shock/MI, and post-op intra-aortic balloon pump (IABP) as potential causes for heterogeneity.

The Cochrane Collaboration principals and the Preferred Reporting Items for Systematic Reviews and Meta-Analyses (PRISMA) guidelines complied with the standards for the current study. Two-tailed levels of 0.05 and 0.1 were set as the limit for the statistical significance of the hypothesis and heterogeneity analyses, respectively. The *p* values were not revised throughout the assessment.

## Results

**Fig. 1:**
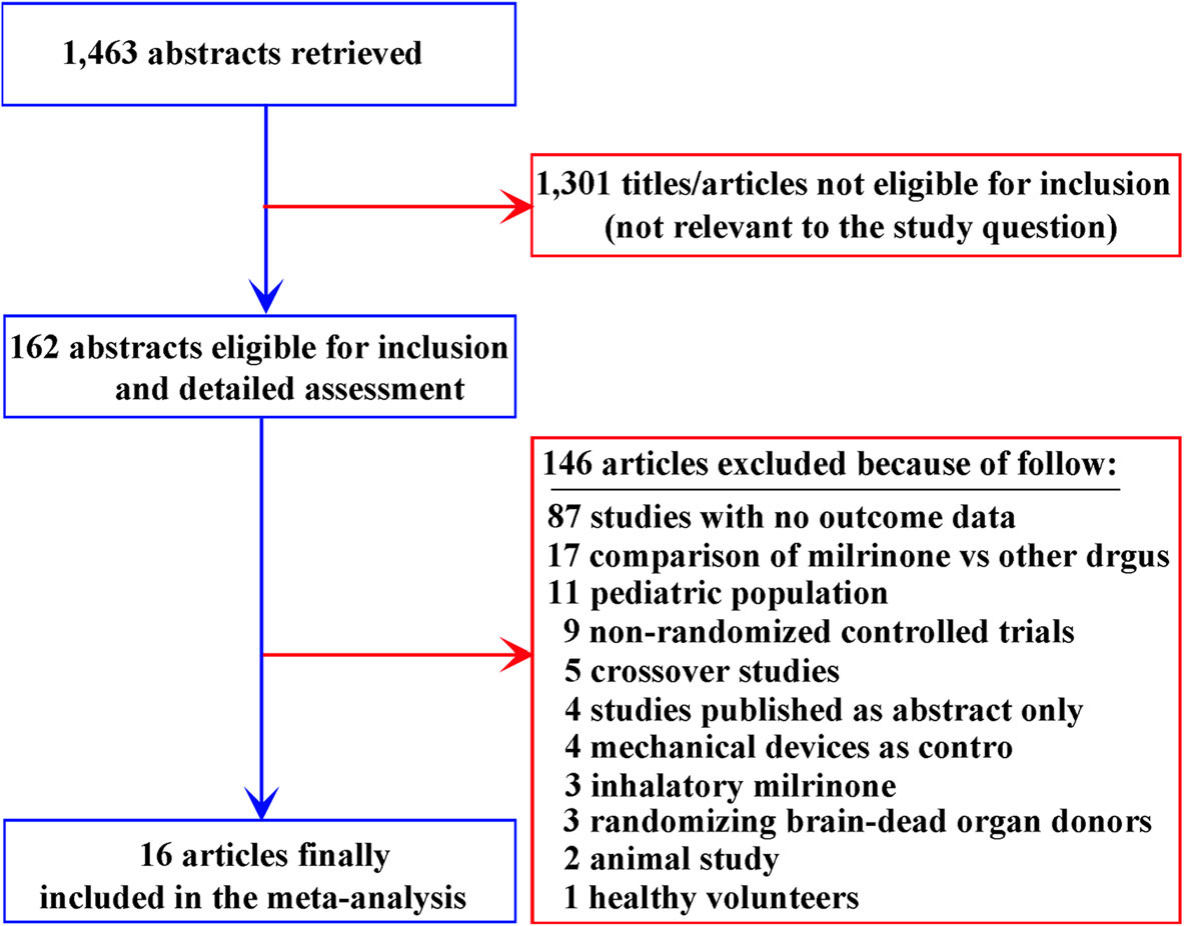
Flow diagram of the study selection

A total of 1463 articles were identified and screened. After the exclusion of 1301 articles due to irrelevant titles or abstracts, 162 full-text studies were eligible and assessed according to the selection criteria (Fig. 1). Of these, the most common reasons for exclusion were as follows: valid data could not be obtained by the authors (87 studies), milrinone was compared with other drugs (17 studies), pediatric populations were used (11 studies), studies were nonrandomized controlled trials (9 studies), crossover studies (5 studies), studies published as abstracts only (4 studies), studies used mechanical devices as controls (4 studies), studies used inhaled milrinone (3 studies), studies used randomization of brain-dead organ donors (3 studies), studies were animal studies (2 studies), and studies used healthy volunteers (1 study). Ultimately, sixteen randomized clinical trials were assessed in compliance with the inclusion criteria (Table 1) [31, 38–47, 52–55].

**Table 1:** A Description of the Studies Included in the Meta-Analysis

First Author	Journal	Year	Procedures	Control	Inclusion Criteria	Exclusion Criteria	Primary Outcome	Secondary Outcome
Arbeus M [38]	Journal of Cardiothoracic and Vascular Anesthesia	2009	Elective CABG	Placebo	Stable angina, LVEF (%) > 30%, Sinus rhythm.	Unspecified	Graft flow	Unspecified
Couture P [39]	Canadian Journal of Anaesthesia	2007	Elective CABG	Placebo	Ischemic heart disease, LV diastolic dysfunction.	Mitral and aortic valvular disease, Atrial fibrillation or pacemaker, Contraindication to TEE.	LV diastolic dysfunction	Unspecified
Doolan LA [40]	Journal of Cardiothoracic and Vascular Anesthesia	1997	Elective CABG and valvular surgery	Placebo	LVEF (%) ≤ 35%, Mean PAP ≥ 20 mmHg.	Supraventricular tachyarrhythmias, Platelet countless than 100 × 109/L (preoperative), Significant primary hepatic or renal disease.	Success or failure in weaning from bypass.	Hemodynamic variables
Guo YJ [9]	Chinese Heart Journal	2014	Elective CABG	Placebo	CABG surgery, LVEF (%) < 35%,	Emergency surgery, Myocardial infarction, Ventricular arrhythmias, Requiring inotropic support.	Myocardial ischemia and/or MI incidence	Mean arterial pressure, Requirements for vasopressors, Death, Length of hospital stay, Serum potassium and creatinine Concentrations.
Hadadzadeh M [41]	Acta medica Iranica	2013	Elective CABG (off-pump)	Placebo	Severe myocardium dysfunction (LVEF (%) < 35%)	Emergency CABG, Myocardial infarction, Ventricular arrhythmias, Requiring inotropic support.	Myocardial ischemia and/or MI incidence	Unspecified
Hamada Y [42]	Japanese circulation journal	1999	Elective CABG and valvular surgery	Standard treatment	Unspecified	Unspecified	Hemodynamic variables	Unspecified
Hayashida N [43]	Annals of Thoracic Surgery	1999	Elective CABG	Standard treatment	Isolated CABG surgery	Myocardial ischemia, Acute myocardial infarction	Cytokine production	Unspecified
Jebeli M [44]	Cardiology Journal	2010	Elective CABG	Placebo	LVEF (%) < 35%,	Emergency CABG, Myocardial infarction, Ventricular arrhythmias, Requiring inotropic support.	Myocardial ischemia and/or MI incidence	Cardiac enzyme levels, Duration of inotropic support.
Jo HR [45]	Korean Journal of Anesthesiology	2010	Elective CABG (off-pump)	Placebo	CABG surgery, Normal LV function.	LVEF or RVEF (%) < 40%, Valvular heart disease, Severe cerebral or renal dysfunction, Emergency surgery.	Right ventricular function and early outcomes	Unspecified
Kwak YL [46]	European journal of cardio-thoracic surgery	2004	Elective CABG (off-pump)	Placebo	Unspecified	Renal or hepatic dysfunction, Thrombocytopenia, Coagulopathy.	Cardiac Index	Unspecified
Lee JH [32]	Journal of korean medical science	2006	Elective CABG (off-pump)	Placebo	RVEF (%) < 35%	Hepatic or renal dysfunction, Thrombocytopenia or coagulopathy, Supraventricular tachyarrhythmias, Single coronary artery disease, History of receiving inotropic agents.	Right ventricular function and early outcomes	Unspecified
Möllhoff T [47]	Anesthesiology	1999	Elective CABG	Placebo	Elective CABG	LVEF (%) < 50%, Gastrointestinal disorders, Diabetes	Splanchnic oxygenation, Systemic inflammation, Subsequent acute-phase response.	Unspecified
Shi YF [48]	Journal of Thoracic and Cardiovascular Surgery	2006	Elective CABG	Placebo	Elective CABG	Had pacemaker or not in sinus rhythm	Biventricular filling properties	Unspecified
Song JW [49]	Korean Journal of Anesthesiology	2011	Elective CABG (off-pump)	Placebo	E/e’ value > 15	Concomitant systolic dysfunction, Mitral regurgitation ≥ Grade 2, Emergency operation.	Hemodynamics and short term outcomes	Unspecified
Yamaguchi A [50]	Annals Of Thoracic And Cardiovascular Surgery	2009	Elective CABG and valvular surgery	Standard treatment	Elective CABG concomitant LVR, LV dysfunction (LVEF (%) < 30%), LVESVI > 100 ml/m^2^	Unspecified	Left ventricular restoration	Unspecified
Yamaura K [51]	Journal of Cardiothoracic and Vascular Anesthesia	2001	Elective CABG	Standard treatment	Cardiac Surgery	Unspecified	Gastric intramucosal pH, Systemic inflammation	Unspecified

*Abbreviations*: *CABG* coronary artery bypass grafting, *LVEF* left ventricular ejection fraction, *TEE* transesophageal echocardiography, *PAP* pulmonary arterial pressure, *AF* atrial fibrillation, *MI* myocardial infarction, *E/e’* the ratio of the early transmitral flow velocity to the early diastolic velocity of the mitral annulus, *LVR* left ventricular restoration, *LVESVI* left ventricular end-systolic volume index

### Study characteristics

**Fig. 2:**
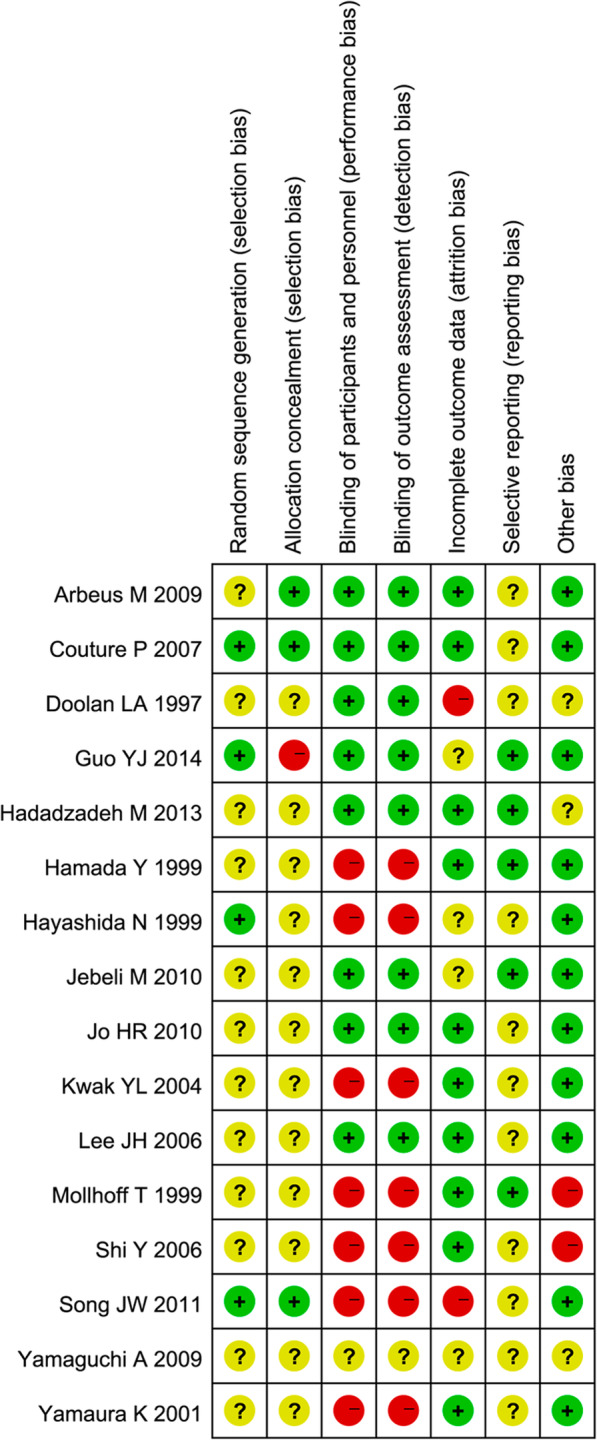
Risk of bias assessment. Review of authors’ judgments about each risk of bias domain for each included study. Red: high risk, green: low risk, yellow: unclear

The total number of patients in the 16 included trials was 698, who underwent CABG surgery (346 treated with placebo/standard care and 352 treated with milrinone) (Table 2 and Table 3). In five of these studies, off-pump CABG surgery was performed [31, 40, 44, 45, 53], and on-pump CABG surgery was performed in 11 studies [38–44, 47, 53, 55, 56]. As the result showed, the mortality of on-pump CABG between milrinone treatment and placebo/standard care groups, the occurrence was [5/139 (3.59%) vs. 4/146 (2.74%), odds ratio (OR) = 1.17 (0.37–3.72), *p* value = 0.649, I-squared = 0.0%]. In contrast, off pump GABG was [6/216 (2.78%) vs. 5/205 (2.44%), odds ratio (OR) = 1.00 (0.14–7.30), *p* value = 1.00, I-squared = 0.0%]. The overall odds ratio (OR) = 1.12 (0.41–3.06), *p* value = 0.869, I-squared = 0.0%. All these results showed that the mortality occurrences were no significantly difference between on-pump and off-pump CABG surgery. The modes of administration included bolus administration [38–42, 44, 47, 53] and continuous infusion [39–41, 48–51, 53–60], which was preceded in 7 studies by an initial bolus [39–41, 44, 47, 53] in which the dose of the bolus varied from 30 to 75 μg/kg, and the dose of the continuous infusion varied from 0.25 to 0.75 μg/kg/min. The quality of the current results was variable. Although 3 RCTs were considered high quality, there were a large number of studies lacking important details needed for evaluating the risk of selection, performance, attrition, or detection biases (Fig. 2).

**Table 2:** Baseline Characteristics and Interventions of Included Trials

Author	Group	Patients	Age	Sex	Time of administration	Milrinone dose		Length of infusion	Duration of follow-up
		(n)	(y)	(M/F)		Bolus	Infusion		
Arbeus M [38]	MIL	22	63 ± 10	20/2	After release of aortic clamp	50 μg/kg	None	Bolus only	Hospital Stay
	Ctrl	22	62 ± 9	17/5					
Couture P [39]	MIL	25	67 ± 8	19/6	After anesthesia induction	50 μg/kg	0.5 μg/kg/min	Until skin closure	Hospital Stay
	Ctrl	25	70 ± 7	19/6					
Doolan LA [40]	MIL	15	65 ± 10.4	14/1	15 min before weaning from CPB	50 μg/kg	0.5 μg/kg/min	4 h or longer	30 day
	Ctrl	15	67 ± 8.6	14/1					
Guo YJ [9]	MIL	31	56 ± 6	21/10	After release of aortic clamp	50 μg/kg	0.5 μg/kg/min	24 h	Hospital Stay
	Ctrl	31	54 ± 6	20/11					
Hadadzadeh M [41]	MIL	40	62 ± 10.7	31/9	After anesthesia induction	50 μg/kg	0.5 μg/kg/min	24 h	ICU Stay
	Ctrl	40	63 ± 9.6	26/14					
Hamada Y [42]	MIL	10	66.2 ± 8.1	6/4	After release of aortic clamp	50 μg/kg	None	Bolus only	Operating Room
	Ctrl	10	62.4 ± 6.5	6/4					
Hayashida N [43]	MIL	12	63.3 ± 2.8	7/5	After anesthesia induction	None	0.5 μg/kg/min	24 h	72 h
	Ctrl	12	62.7 ± 2.8	9/3					
Jebeli M [44]	MIL	35	56.9 ± 9.7	25/10	After release of aortic clamp	50 μg/kg	0.5 μg/kg/min	24 h	Hospital Stay
	Ctrl	35	58.2 ± 8.4	28/7					
Jo HR [45]	MIL	20	67.0 ± 9.2	12/8	After sternotomy	None	0.5 μg/kg/min	Until skin closure	Hospital Stay
	Ctrl	20	64.1 ± 9.9	11/9					
Kwak YL [46]	MIL	29	61.5 ± 8.2	21/8	After IMA harvest	None	0.5 μg/kg/min	End of anastomosis	Hospital Stay
	Ctrl	33	60.4 ± 8.4	26/7					
Lee JH [32]	MIL	24	63 ± 8	20/4	After sternotomy	None	0.5 μg/kg/min	Until skin closure	Hospital Stay
	Ctrl	26	62 ± 8	20/6					
Möllhoff T [47]	MIL	11	60 ± 8	Not specified	After anesthesia induction	30 μg/kg	0.5 μg/kg/min	Unspecified	1 Year
	Ctrl	11	61 ± 6						
Shi Y [53]	MIL	25	Not specified	Not specified	After anesthesia induction	50 μg/kg	0.5 μg/kg/min	Until skin closure	6 month
	Ctrl	24				High: 75 μg/kg	High: 0.75 μg/kg/min		
Song JW [54]	MIL	31	67.2 ± 7.6	14/17	After harvesting the left	None	0.5 μg/kg/min	31 ± 7 min	Hospital Stay
	Ctrl	31	65.7 ± 7.9	21/10	internal mammary artery				
Yamaguchi A [55]	MIL	14	64.1 ± 8	13/1	After induction of CPB	None	0.5 μg/kg/min	48 h	ICU Stay
	Ctrl	14	65.2 ± 8.5	13/1					
Yamaura K [56]	MIL	10	66 ± 6	7/3	After induction of CPB	None	0.25 μg/kg/min	1 h after in ICU	Hospital Stay
	Ctrl	10	57 ± 16	6/4

**Table 3:** Preoperative Ejection Fraction and Postoperative Causes of Death in the 2 Groups

First Author	Preoperative EF (MIL Group)	Preoperative EF (Ctrl Group)	No. of Death (Death/Total, MIL Group)		No. of Death (Death/Total, Ctrl Group)		Cause of Death (MIL Group)	Cause of Death (Ctrl Group)	Adverse Effects (MIL Group)	Adverse Effects (Ctrl Group)
Arbeus [38]	59 ± 12	63 ± 9	1	22	0	22	Not specified	No death	No adverse events or side effects	
**Couture** [39]	51 ± 15	50 ± 13	2	25	0	25	Multiple organ failure (2)	No death	Acute renal failure (2)	Acute renal failure (1)
**Doolan** [40]	Not specified	Not specified	0	15	0	15	No death	No death	No adverse events or side effects	
**Guo** [9]	35 ± 4	35 ± 5	1	31	1	31	Not specified	Not specified	Not specified	
**Hadadzadeh** [41]	29 ± 5.5	28.6 ± 5.6	1	40	1	40	Cardiac shock	Cardiac shock	CVA, Renal failure (1)	CVA, Renal failure (3)
**Hamada** [42]	Not specified	Not specified	0	20	0	10	No death	No death	No adverse events or side effects	
**Hayashida** [43]	Not specified	Not specified	0	12	0	12	No death	No death	No serious adverse effects	Low output syndrome (1)
**Jebeli** [44]	31.8 ± 3.2	34.5 ± 1.4	0	35	2	35	No death	Cardiogenic shock (2)	No adverse events or side effects	
**Jo** [45]	45 ± 14	51 ± 13	0	20	0	20	No death	No death	Renal failure (1)	Renal failure (2)
**Kwak** [46]	Not specified	Not specified	0	29	0	33	No death	No death	No adverse events or side effects	
**Lee** [32]	50 ± 17	57 ± 8	0	24	0	26	No death	No death	No adverse events or side effects	
**Möllhoff** [47]	Not specified	Not specified	0	11	0	11	No death	No death	No adverse events or side effects	
**Shi** [53]	Not specified	Not specified	1	25	1	24	Not specified	Not specified	Not specified	
**Song** [54]	55.3 ± 15.3	51.5 ± 16.7	1	31	1	31	Not specified	Not specified	Not specified	
**Yamaguchi** [55]	64.1 ± 8	65.2 ± 8.5	0	14	0	14	No death	No death	Not specified	
**Yamaura** [56]	Not specified	Not specified	0	10	0	10	No death	No death	No adverse events or side effects

### Quantitative data synthesis

**Fig. 3:**
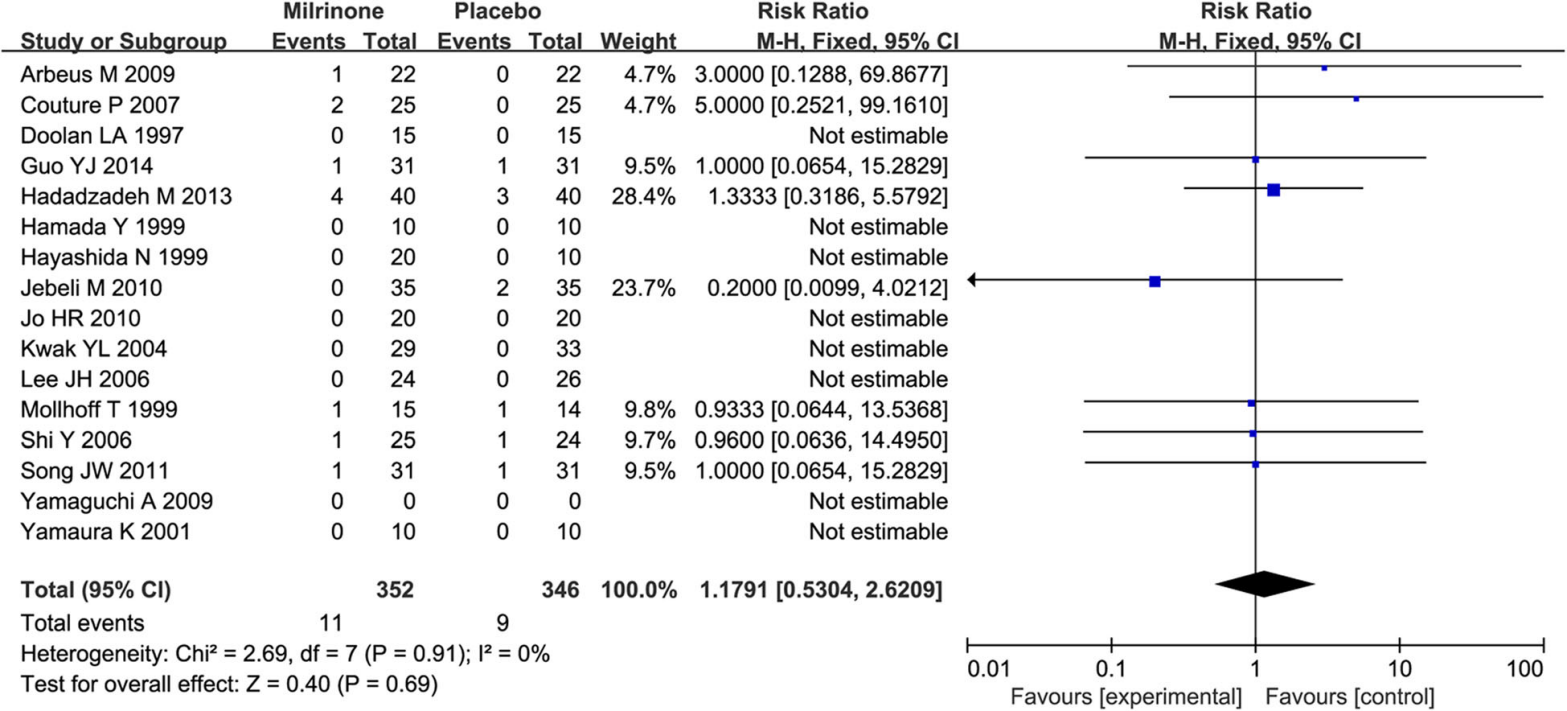
A forest plot of the risk of mortality. CI, confidence interval; df, degrees of freedom

The overall analysis demonstrated that the mortality rate was not higher in patients receiving milrinone than in patients receiving placebo/standard care [11/352 (3.13%): mortality in the milrinone treatment group 9/346 (2.60%) versus mortality in the control group, RR = 1.18 (0.53–2.62), *p* value = 0.69, *p* for heterogeneity = 0.91, I^2^ = 0%] (Fig. 3).

Sensitivity analysis and funnel plot inspection confirmed the overall robustness of the present findings and the lack of evidence of small-study bias, respectively (Fig. 5**a**).

**Fig. 4:**
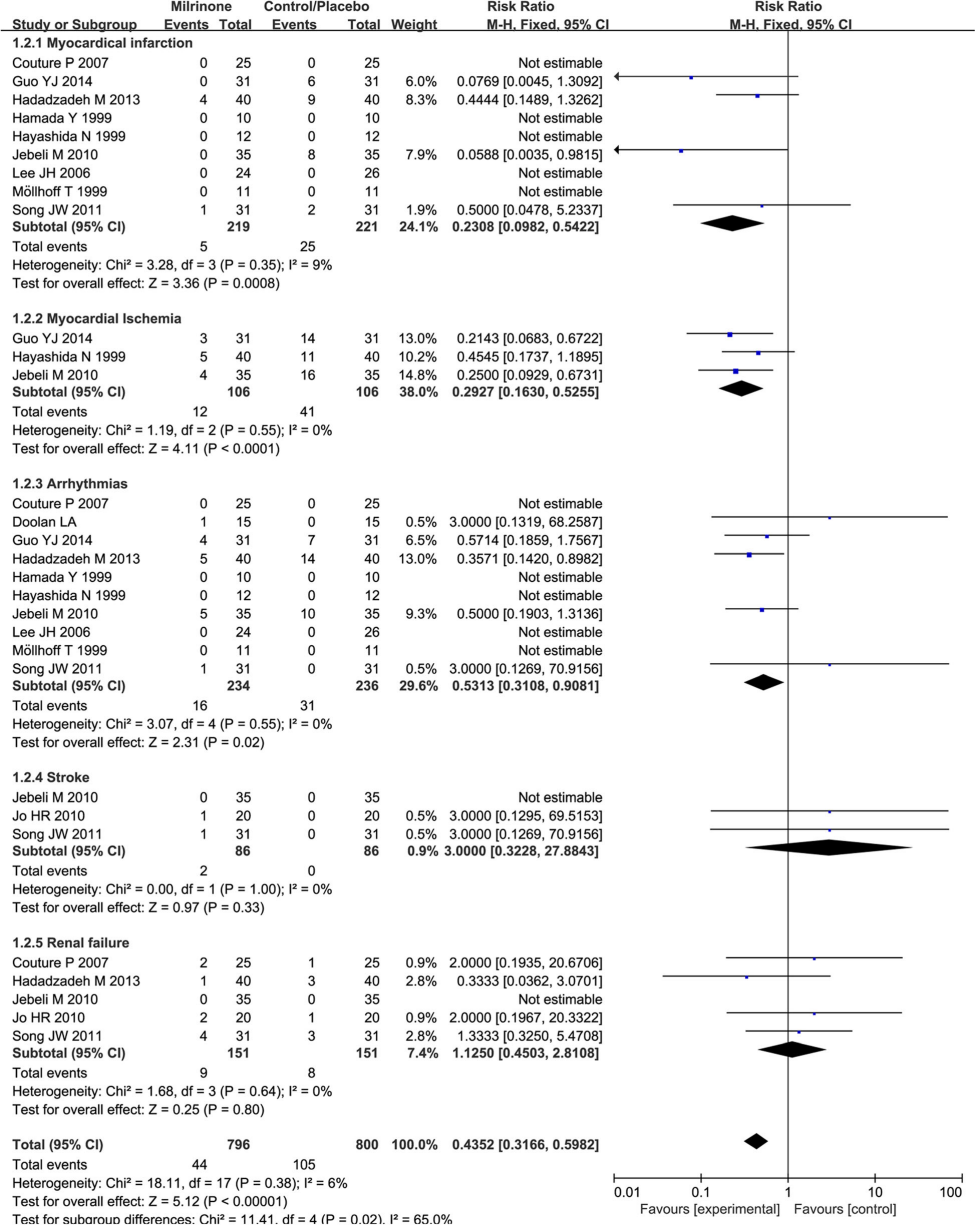
Forest plot of all-cause mortality in trials stratified by intervention

The subanalysis of different postoperative outcomes (Fig. 4**,** Table 4) showed a statistically significant effect of milrinone on reducing the occurrence of MI [5/219 (2.28%) in the milrinone treatment group versus 25/221 (11.31%) in the control group, RR = 0.23 (0.10–0.54), *p* value = 0.0008, *p* for heterogeneity = 0.35, I^2^ = 9%, with 9 studies included], myocardial ischemia [12/106 (11.32%) in the milrinone treatment group vs. 41/106 (36.68%) in the control group, RR = 0.29 (0.16–0.52), *p* value < 0.0001, *p* for heterogeneity = 0.55, I^2^ = 0%, with 3 studies included], and arrhythmia [16/234 (6.84%) in the milrinone treatment group vs. 31/236 (13.14%) in the control group, RR = 0.53 (0.31–0.91), *p* value = 0.02, *p* for heterogeneity = 0.55, I^2^ = 0%, with 10 studies included].

**Table 4:** A Summary of the Global Effect of Different Outcomes

	Patients (Studies) Included	Milrinone: Events (%)	Control: Events (%)	RR	95% CI	***p*** for Effect	I^**2**^ (***p*** for heterogeneity)
Myocardical Infarction	440 (30)	5 (2.28%)	25 (11.31%)	0.23	0.10–0.54	0.0008	9% (0.35)
Myocardial Ischemia	212 (53)	12 (11.32)	41 (36.68)	0.29	0.16–0.52	< 0.0001	0% (0.55)
Arrhythmias	470 (47)	16 (6.84)	31 (13.14)	0.53	0.31–0.91	0.02	0% (0.55)
Stroke	172 (2)	2 (2.33)	0 (0)	3.00	0.32–27.88	0.33	0% (1.00)
Renal Failure	302 (17)	9 (5.96)	8 (5.30)	1.25	0.45–2.81	0.80	0% (0.64)

**Fig. 5:**
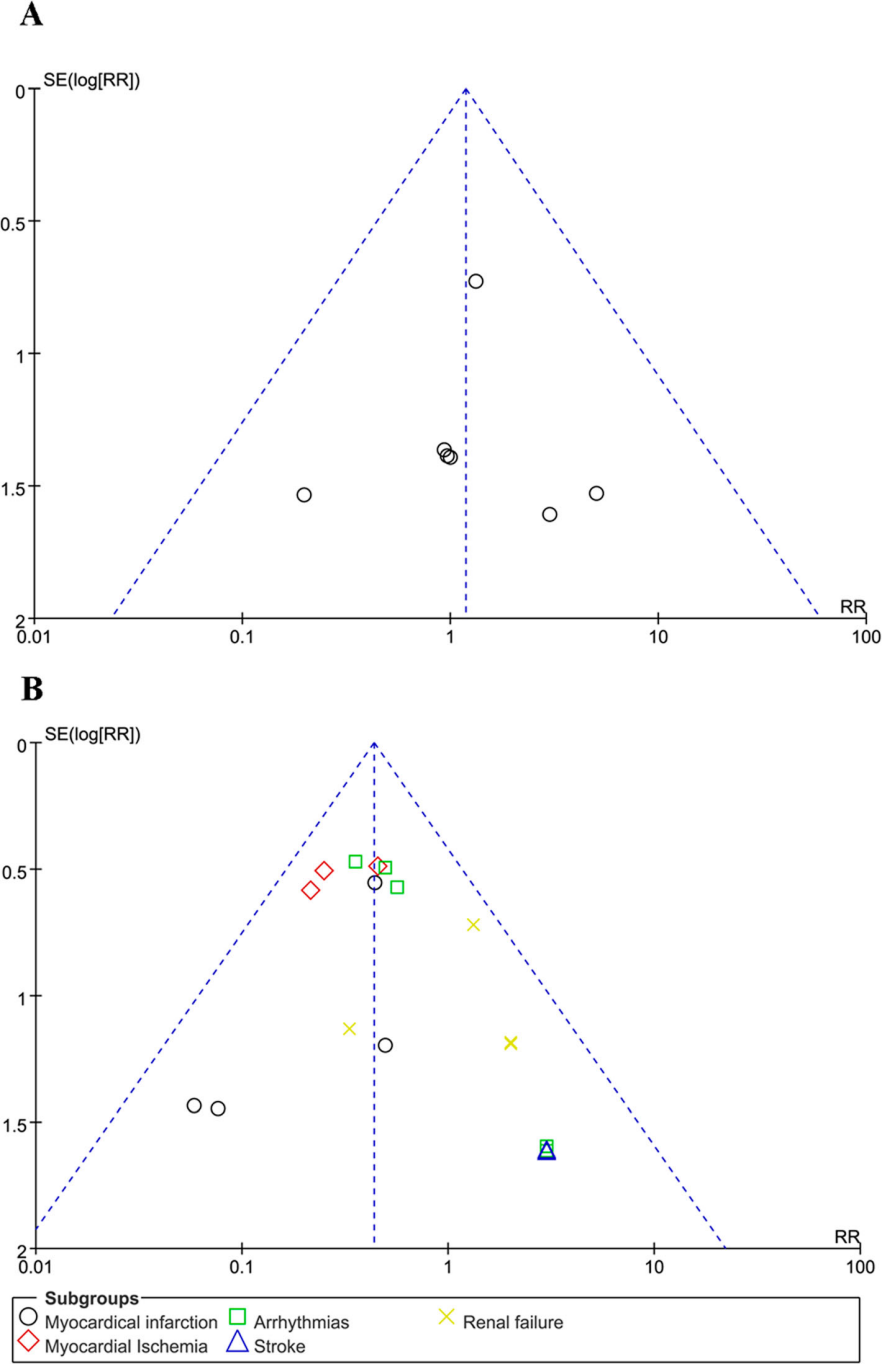
A funnel plot of the risk of mortality. SE, standard error. **a**. **b**

Another subanalysis showed a difference in the risk of stroke [2/86 (2.33%) in the milrinone treatment group vs. 0/86 (0%) in the control group, RR = 3.00 (0.32–27.88), *p* value = 0.33, *p* for heterogeneity = 1.00, I^2^ = 0%, with 3 studies included] and renal failure [9/151 (5.96%) in the milrinone treatment group vs. 8/151 (5.30%) in the control group, RR = 1.25 (0.45–2.81), *p* for effect = 0.80, *p* for heterogeneity = 0.64, I^2^ = 0%, with 5 studies included]. Sensitivity analysis and funnel plot inspection confirmed the overall robustness of the present findings and the lack of evidence of small-study bias, respectively (Fig. 5b).

### Meta-regression

Meta-regression was used to analyze potential causes for heterogeneity on one-year mortality. The results showed that there were not statistically significant for sample size, mean number of grafts, mean pump time, mean AoXclamp time, mean pre-op LVEF, post-op inotropes, pre-op shock/MI, and post-op IABP (Tables 5 and 6). All these parameters were not associated with mortality.

**Table 5:** The meta-regression analyses

Parameter	Regression	P value
Sample size	y = 0.0022x + 0.0603	0.9488
Mean number of grafts	y = −0.0368x + 0.2930	0.9926
Mean pump time	y = 0.0092x + 1.0395	0.6035
Mean AoXclamp time	y = −0.0091x + 0.9190	0.6505
Mean preop LVEF	y = 0.0160x - 0.4410	0.6420
Postop inotropes	y = −0.0629x + 1.3785	0.2812
Preop shock/MI	y = −0.0263x + 0.2877	0.7811
Postop IABP	y = −0.1269x - 0.7856	0.5557

**Table 6:** The meta-regression analyses of preop drugs

Preop drugs	Regression	P value
ACE inhibitors	y = − 0.0713x + 1.3446	0.6860
Calcium channel inhibitors	y = − 0.0396x + 1.1287	0.6836
Diuretics	y = − 0.0066x + 0.0849	0.9734
Nitrates	y = 0.0997x - 0.6147	0.6246
β-receptor inhibitors	y = 0.0128x + 0.2710	0.8870

## Discussion

In this study, we conducted a systematic meta-analysis of all existing, enrolled and randomized studies comparing milrinone treatment to placebo/standard care in patients who underwent CABG surgery. The results showed that compared with placebo treatment, milrinone treatment did not contribute to mortality. Although milrinone failed to reduce mortality, the risk of postoperative complications, such as MI, myocardial ischemia, and arrhythmia, was significantly decreased when patients underwent CABG surgery.

Approximately 110 million people are affected by CAD, which resulted in 8.9 million deaths in 2015 [56]. CAD is considered the most common cause of death globally because of its high mortality risk (15.9%) [57]. From 1980 to 2010, the number of cases and the risk of death from CAD for a given age both declined, especially in developed countries [58, 59]. Some well-determined risk factors, including high blood pressure, smoking, diabetes, obesity, family history, and excessive alcohol, were controlled. Approximately half of the cases result from genetics among all these factors [48, 49, 60]. Obesity and smoking are associated approximately 20 and 36% of cases, respectively [50]. The typical pathophysiological characteristic of CAD is limited blood flow to the heart, which may result in ischemia and long-term oxygen deficiencies in heart muscle, leading to cell death and, ultimately, causing myocardial infarction (MI). In addition, transient ischemia resulting from coronary artery stenosis may lead to ventricular arrhythmia, devolve into a dangerous heart rhythm, and lead to death, which is known as ventricular fibrillation [51]. Although a Cochrane review in 2015 suggested that combining preventive strategies such as persisting appropriate physical exercise, maintaining a healthy diet, treating hypertension, reducing cholesterol and quitting smoking could effectively prevent the risk of CAD [61–65]. there was insufficient evidence to prove an impact on mortality or actual cardiovascular events [66]. Until now, the most effective treatment options for moderate to severe CAD have been medications (such as statins, nitroglycerin, calcium channel blockers, and/or beta-blockers and aspirin) [67–69] and surgery (such as CABG surgery) [70–72]. CABG surgery is performed to treat coronary artery disease (CAD) by using a grafted vein to establish vascular access between the root of the ascending aorta and the distal end of the lesion site so that blood can bypass the coronary artery lesion site and reach the ischemic myocardium, thus improving coronary perfusion and increasing myocardial oxygen supply, which is also called myocardial revascularization [73, 74]. Numerous studies have demonstrated that CABG surgery is associated with low mortality (in both the short term and the long term) as well as cognitive and renal function benefits [75, 76]. In the past decade, percutaneous coronary intervention (PCI) has been increased to treat unprotected left main coronary artery disease. PCI can be selectively performed in patients who are candidates for revascularization but who are ineligible for CABG. Compared with CABG, PCI with stenting has a similar mortality but higher rates of myocardial infarction and repeat revascularisation in patients with left main coronary artery disease. So current guidelines recommend CABG as the treatment of choice for patients with asymptomatic ischemia, stable angina, or unstable angina/non-ST elevation myocardial infarction who have left main coronary artery disease [77, 78]. However, multiple complications (including MI, myocardial ischemia, arrhythmia, stroke, and kidney failure) are common postoperative syndromes after CABG [7, 17, 19, 20, 79]. Surgery, combined with medication pre- and/or postoperatively, such as inotropic agents, which can increase myocardial contractility that results, in most cases, in increasing intracellular cAMP levels, can effectively avoid or ameliorate these unwanted outcomes [80–82]. Increased cAMP subsequently stimulates adenylate cyclase and inhibits PDE III simultaneously [83]. Despite (or because of) their effectiveness, inotropic agents face various substantial limitations, such as acute myocardial β-adrenergic receptor desensitization, limiting the function for post-bypass cardiac failure [84]; additional observational data suggest that inotropic agents contribute to worse clinical outcomes due to the high incidence of renal dysfunction and death ratio [85–88].

PDE III inhibitors such as milrinone provide an alternative option to inotropic support [84] because they have not only positive inotropic effects but also vasodilatory effects [83, 89]. The preemptive use of milrinone has been beneficial for renal tubular injury [85]. Unlike dobutamine, milrinone does not increase heart rate or myocardial oxygen consumption [90], and some studies have reported that milrinone can significantly reduce the risk of postoperative myocardial ischemia and infarction in patients undergoing CABG surgery [43]. However, one of the current controversies or unknown questions in terms of milrinone application is whether the drug is associated with mortality. A recent meta-analysis by Zangrillo A et al. [34] showed that compared with control agents, milrinone had a tendency to increase mortality and the incidence of arrhythmia in patients who underwent cardiac surgery [13/249 (5.2%) in milrinone vs. 6/269 (2.2%) in the control arm, OR = 2.67 (1.05–6.79), *p* for effect = 0.04, *p* for heterogeneity = 0.23, I^2^ = 25%). However, in their study, 13 trials were included that involved different control agents (3 with levosimendan, 2 with nesiritide, 7 with placebo, and 1 with nothing). These factors may have induced a bias risk. For instance, a subanalysis with placebo or nothing as a control demonstrated no difference in the risk of mortality [4/165 (2.4%) with milrinone vs. 3/164 (1.8%) in the control arm, OR = 1.27 (0.28–5.84), *p* for effect = 0.76, *p* for heterogeneity = 0.45, I^2^ = 0%, 329 patients and 8 studies included]. In addition, an updated meta-analysis [34] showed that neither the overall nor the subgroup (adult patients) mortality in the milrinone-treated group was significantly different from that in the control group (mortality, 2.2% vs. 2.1%, *p* = 0.70 overall, 3% vs. 2.4%, *p* = 0.70 in adult patients). However, the sensitivity analysis with a low risk of bias showed a trend, but not statistical significance, toward an increase in mortality with milrinone [8/153 (5.2%) in the milrinone arm vs. 2/152 (1.3%) in the control arm, RR = 2.71 (0.82–9), *p* for effect = 0.10]. Furthermore, the most recent studies published in 2015 [91] and 2016 [92] demonstrated that there were no differences in mortality in patients administered milrinone compared to the control groups. All these reasons may induce a bias risk.

To avoid these interference factors, we enrolled 16 trials with a randomized total of 698 patients undergoing CABG surgery (346 treated with placebo or standard care and 352 treated with milrinone); the results showed that there was no difference in mortality between the group receiving milrinone and the placebo/standard care group. Nevertheless, the subanalysis demonstrated that the occurrence of myocardial infarction, myocardial ischemia, and arrhythmia decreased significantly with milrinone treatment compared to the placebo or standard care group. However, the occurrence of stroke and renal failure, need for IABP, and duration of inotropic support (h) and mechanical ventilation (h) between these two groups showed no differences. Milrinone was introduced as an agent that causes reduced left and right heart-filling pressures due to its greater reduction in vascular resistance, and it has been used in the treatment of low cardiac output syndrome following cardiac surgery. In the meta-analysis of patients with myocardial infarction suffering from CABG surgery, milrinone was used at any dose and administration time. Mortality after milrinone treatment was not improved despite reductions in important cardiovascular (CV) endpoints. Although the results and conclusions were associated with those of other studies, there may be several reasons for the presented results. First, the association between bias risk and estimates of intervention effects was ignored. Second, the number of included patients was still far too small to draw any firm conclusions. Third, the indications for CABG surgery are relatively extensive. We did not classify the causes of CABG surgery in detail. Therefore, in future studies, additional trial details need to be considered.

Although the evidence in the present study demonstrated that milrinone failed to show an advantage in mortality in adult CABG patients, it significantly reduced the occurrence of MI, myocardial ischemia, and arrhythmia compared to the placebo. All these findings may be helpful for the clinical application of milrinone and may provide therapeutic strategies for CABG surgery. Furthermore, along with clinical milrinone application, sufficient data from randomized clinical trials need to be collected, and the potential benefits or adverse effects should be analyzed and reevaluated.

### Limitations

Our study has several limitations. First, the authors acknowledge that only 4 of the 16 studies included in this meta-analysis were of high quality. Second, in the enrolled RCTs, the doses of milrinone were between 30 and 75 μg/kg (as an intravenous bolus) and between 0.5 and 0.75 μg/kg/min (as continuous infusion). This fact suggests that the current reference lacks generalizability of milrinone at doses beyond the range of 0.3–0.75 μg/kg/min. Third, our study on the incidence of myocardial ischemia, stroke, and renal failure was performed using a small number of studies and patients. Therefore, the current results are not conclusive due to the possibility of induced error. Finally, only one trial was evaluated with a 1-year follow-up, so deficits in the short follow-up could have potentially impacted our mortality analyses.

## Conclusions

This meta-analysis suggests that, compared to placebo or standard care, milrinone neither significantly increases nor decreases the risk of dying in adult patients undergoing CABG surgery, but milrinone can efficiently ameliorate the incidence of postoperative complications, including MI, myocardial ischemia, and arrhythmia.

## Data Availability

The dataset supporting the conclusions of this article is included within the article.

## Abbreviations

AKI: Acute kidney injury
ARF: Acute renal failure
CABG: Coronary artery bypass graft
CAD: Coronary artery disease
cAMP: Cyclic adenosine phosphate
CVDs: Cardiovascular diseases
LOS: Low output syndrome
MI: Myocardial infarction
WHO: World Health Organization
PDE: Phosphodiesterase
RCTs: Randomized controlled trials
PRISMA: Preferred reporting items for systematic reviews and meta-analyses
LVEF: Left ventricular ejection fraction
AKI: Acute kidney injury
IABP: Intra-aortic balloon pump
